# Oral cholera vaccination in hard-to-reach communities, Lake Chilwa, Malawi

**DOI:** 10.2471/BLT.17.206417

**Published:** 2018-09-27

**Authors:** Francesco Grandesso, Florentina Rafael, Sikhona Chipeta, Ian Alley, Christel Saussier, Francisco Nogareda, Monica Burns, Pauline Lechevalier, Anne-Laure Page, Leon Salumu, Lorenzo Pezzoli, Maurice Mwesawina, Philippe Cavailler, Martin Mengel, Francisco Javier Luquero, Sandra Cohuet

**Affiliations:** a Epicentre, 8 rue Saint-Sabin, 75011 Paris, France.; b Agence de Médecine Préventive, Paris, France.; c Directorate of Preventive Health Services, Ministry of Health, Lilongwe, Malawi.; d Department of Infectious Hazard Management, World Health Organization, Geneva, Switzerland.; e Médecins Sans Frontières, Paris, France.

## Abstract

**Objective:**

To evaluate vaccination coverage, identify reasons for non-vaccination and assess satisfaction with two innovative strategies for distributing second doses in an oral cholera vaccine campaign in 2016 in Lake Chilwa, Malawi, in response to a cholera outbreak.

**Methods:**

We performed a two-stage cluster survey. The population interviewed was divided in three strata according to the second-dose vaccine distribution strategy: (i) a standard strategy in 1477 individuals (68 clusters of 5 households) on the lake shores; (ii) a simplified cold-chain strategy in 1153 individuals (59 clusters of 5 households) on islands in the lake; and (iii) an out-of-cold-chain strategy in 295 fishermen (46 clusters of 5 to 15 fishermen) in floating homes, called *zimboweras*.

**Finding:**

Vaccination coverage with at least one dose was 79.5% (1153/1451) on the lake shores, 99.3% (1098/1106) on the islands and 84.7% (200/236) on *zimboweras*. Coverage with two doses was 53.0% (769/1451), 91.1% (1010/1106) and 78.8% (186/236), in the three strata, respectively. The most common reason for non-vaccination was absence from home during the campaign. Most interviewees liked the novel distribution strategies.

**Conclusion:**

Vaccination coverage on the shores of Lake Chilwa was moderately high and the innovative distribution strategies tailored to people living on the lake provided adequate coverage, even among hard-to-reach communities. Community engagement and simplified delivery procedures were critical for success. Off-label, out-of-cold-chain administration of oral cholera vaccine should be considered as an effective strategy for achieving high coverage in hard-to-reach communities. Nevertheless, coverage and effectiveness must be monitored over the short and long term.

## Résumé

**Objectif:**

Évaluer la couverture vaccinale, identifier les raisons de non-vaccination et estimer le degré de satisfaction vis-à-vis de deux stratégies innovantes de distribution de la seconde dose vaccinale, dans le cadre d'une campagne de vaccination orale anticholérique menée en 2016 sur le lac Chilwa et ses environs, au Malawi, en réponse à une flambée de choléra.

**Méthodes:**

Nous avons réalisé une enquête en grappes à deux degrés. La population interrogée a été divisée en trois strates, en fonction de la stratégie de distribution employée pour la seconde dose du vaccin: (i) une stratégie standard pour 1477 personnes (68 grappes de 5 ménages) résidant en bordure du lac; (ii) une stratégie de chaîne du froid simplifiée pour 1153 personnes (59 grappes de 5 ménages) résidant sur des îles du lac; et (iii) une stratégie sans chaîne du froid pour 295 pêcheurs (46 grappes de 5 à 15 pêcheurs) vivant dans des maisons flottantes appelées *zimboweras*.

**Résultats:**

La couverture vaccinale avec administration d'au moins une dose de vaccin a été de 79,5% (1153/1451) dans la population du rivage du lac, de 99,3% (1098/1106) dans la population des îles et de 84,7% (200/236) chez les habitants des *zimboweras*. Dans ces trois strates, la couverture vaccinale avec deux doses a été respectivement de 53,0% (769/1451), 91,1% (1010/1106) et 78,8% (186/236). La raison la plus courante de non-vaccination a été l'absence du domicile durant la campagne. La plupart des personnes interrogées ont apprécié les nouvelles stratégies de distribution.

**Conclusion:**

La couverture vaccinale sur les rives du lac Chilwa a été modérément élevée, et les stratégies innovantes de distribution spécifiquement adaptées pour les personnes vivant sur le lac ont permis une couverture adéquate, y compris parmi les populations difficiles à atteindre. L'implication de la communauté et l'utilisation de procédures simplifiées de distribution ont été des facteurs déterminants de succès. L'administration hors AMM, sans chaîne du froid, de vaccins oraux anticholériques devrait être considérée comme une stratégie efficace pour obtenir une couverture vaccinale élevée dans les communautés difficiles à atteindre. Néanmoins, la couverture et son efficacité doivent être surveillées à court et à long termes.

## Resumen

**Objetivo:**

Evaluar la cobertura de vacunación, identificar los motivos de la no vacunación y evaluar la satisfacción con dos estrategias innovadoras para la distribución de segundas dosis en una campaña de vacunación oral contra el cólera en 2016 en el Lago Chilwa, Malawi, en respuesta a un brote de cólera.

**Métodos:**

Se llevó a cabo una encuesta de conglomerados en dos etapas. La población entrevistada se dividió en tres estratos de acuerdo con la estrategia de distribución de la segunda dosis de la vacuna: (i) una estrategia estándar en 1477 individuos (68 grupos de 5 hogares) a orillas del lago; (ii) una estrategia simplificada de la cadena de frío en 1153 individuos (59 grupos de 5 hogares) en las islas del lago; y (iii) una estrategia fuera de la cadena de frío en 295 pescadores (46 grupos de 5 a 15 pescadores) en hogares flotantes, llamados zimboweras.

**Resultados:**

La cobertura de vacunación con al menos una dosis fue del 79,5 % (1153/1451) en las orillas del lago, del 99,3 % (1098/1106) en las islas y del 84,7 % (200/236) en las zimboweras. La cobertura con dos dosis fue del 53,0 % (769/1451), del 91,1 % (1010/1106) y del 78,8 % (186/236) en los tres estratos, respectivamente. La razón más común para no vacunarse fue estar ausentes del hogar durante la campaña. A la mayoría de los entrevistados les gustaron las nuevas estrategias de distribución.

**Conclusión:**

La cobertura de vacunación a las orillas del lago Chilwa fue moderadamente alta y las innovadoras estrategias de distribución adaptadas a las personas que viven en el lago proporcionaron una cobertura adecuada, incluso entre las comunidades de difícil acceso. La participación de la comunidad y la simplificación de los procedimientos de administración fueron fundamentales para el éxito. La administración de la vacuna oral contra el cólera sin receta y fuera de la cadena de frío debería considerarse una estrategia eficaz para lograr una cobertura alta en comunidades de difícil acceso. No obstante, la cobertura y la eficacia deben ser objeto de seguimiento a corto y largo plazo.

## ملخص

**الغرض:**

تقييم التغطية التي يقدمها التطعيم، وتحديد أسباب عدم التطعيم وتقييم مدى الرضا من خلال استراتيجيتين مبتكرتين لتوزيع الجرعات الثانية في حملة لقاح الكوليرا الفموية في عام 2016 في ليك شيلوا بملاوي، كرد فعل بعد تفشي الكوليرا.

**الطريقة:**

أجرينا مسحا مجمعاً يتكون من مرحلتين. تم تقسيم السكان الذين تمت مقابلتهم إلى ثلاث طبقات وفقًا لاستراتيجية توزيع لقاح الجرعة الثانية: استراتيجية قياسية لعدد 1477 فردًا (68 مجموعة من 5 أسر) على شواطئ البحيرة؛ و(2) استراتيجية مبسطة لسلسلة التبريد لعدد 1153 فرداً (59 مجموعة من 5 أسر) في جزر البحيرة؛ و(3) استراتيجية لسلسلة من خارج التبريد لعدد 295 صياداً (46 مجموعة من 5 إلى 15 صياداً) في منازل عائمة تسمى *زيمبوراز *.

**النتائج:**

وصلت نسبة التغطية بجرعة واحدة على الأقل التي يقدمها التطعيم إلى 79.5٪ (1153/1451) على شواطئ البحيرة، وبلغت 99.3٪ (1098/1106) في الجزر، و84.7٪ (200/236) في منازل *زيمبوراز * العائمة. بلغت التغطية بجرعتين 53.0٪ (769/1451)، و91.1٪ (1010/1106)، و78.8٪ (186/236)، في الطبقات الثلاث على التوالي. كان السبب الأكثر شيوعا لعدم التطعيم هو الغياب عن المنزل أثناء الحملة. وأعرب معظم من أجريت معهم المقابلات عن إعجابهم باستراتيجيات التوزيع المبتكرة.

**الاستنتاج:**

كانت التغطية التي يقدمها التطعيم على شواطئ ليك تشيلوا عالية إلى حد ما، وقدمت استراتيجيات التوزيع المبتكرة، المصممة للأشخاص الذين يعيشون في البحيرة تغطية كافية، حتى بين المجتمعات التي يصعب الوصول إليها. كانت مشاركة المجتمع والإجراءات المبسطة لطرح التطعيم، عوامل حاسمة بالنسبة للنجاح. إن تقديم اللقاح الفموي من الكوليرا بدون تسمية من خارج سلسلة التبريد، ينبغي اعتباره كاستراتيجية فعالة لتحقيق تغطية عالية في المجتمعات التي يصعب الوصول إليها. ومع ذلك، فإنه يجب مراقبة التغطية والفعالية على المدى القصير والطويل. ****

## 摘要

**目的:**

旨在评估疫苗接种覆盖率、确定未接种疫苗的原因并评估对两项创新策略的满意度。这两项创新策略是针对霍乱疫情爆发、为 2016 年马拉维共和国奇尔瓦湖一次口服霍乱疫苗运动中的第二剂疫苗分发而制定。

**方法:**

我们执行了双阶段类集调查。基于第二剂疫苗分发策略，将受访人群分为三个层级：(i) 覆盖湖岸 1477 人（68 个群组，以 5 户为一单位）的标准策略；(ii) 覆盖湖中岛屿 1153 人（59 个群组，以 5 户为一单位）的简化冷链策略；(iii) 覆盖水上房屋（亦称 *zimboweras*) 295 名渔民（46 个群组，5 至 15 名渔民为一单位）的冷链外策略。

**结果:**

至少接种一剂疫苗的覆盖率分别是湖岸：79.5％(1153/1451)，岛屿：99.3％(1098/1106) 和水上房屋 (*zimboweras*)：84.7％(200/236)。三个层级中，接种两剂疫苗的覆盖率分别为 53.0％(769/1451)，91.1％(1010/1106) 和 78.8％(186/236)。人们未接种疫苗的最常见原因是疫苗运动期间离家，从而没有参与。大多数受访者都倾向于创新分发策略。

**结论:**

奇尔瓦湖岸边的疫苗接种覆盖率较高。同时，即便是在难以触及的社区，为湖上居民量身定制的创新分发策略也达到了足够的覆盖率。社区参与和简化交付流程对此次成功至关重要。对口服霍乱疫苗标签外、无冷链外的管理应被视为一项在难以触及的社区实现高覆盖率的有效战略。然而，对覆盖率和有效性的短期、长期监测是必不可少的。

## Резюме

**Цель:**

Оценить покрытие вакцинацией, выявить причины отсутствия вакцинации и оценить удовлетворенность двумя инновационными стратегиями распределения второй дозы вакцинации пероральной холерной вакцины в рамках кампании, проводившейся в 2016 году в районе озера Чилва (Малави) в ответ на вспышку холеры в регионе.

**Методы:**

Авторы провели двухэтапный кластерный опрос. Опрашиваемое население делилось на три группы согласно стратегиям распределения второй дозы вакцины: (i) стандартная стратегия для 1477 человек (68 кластеров 5 семейств) на побережье озера; (ii) упрощенная стратегия холодовой цепи для 1153 человек (59 кластеров 5 семейств) на островах; (iii) не предусматривающая холодовой цепи стратегия для 295 рыбаков (46 кластеров численностью от 5 до 15 рыбаков) в плавучих домах, так называемых *зимбоверах*.

**Результаты:**

Количество людей, принявших хотя бы одну дозу вакцины, составило 79,5% (1153/1451) на побережье озера, 99,3% (1098/1106) на островах и 84,7% (200/236) в *зимбоверах*. Охват двумя дозами в этих трех группах составил 53,0% (769/1451), 91,1% (1010/1106) и 78,8% (186/236) соответственно. Наиболее частой причиной невакцинирования было отсутствие дома в момент проведения кампании. Большинству опрошенных новые стратегии распределения вакцины понравились.

**Вывод:**

Вакцинирование областей, расположенных на берегах озера Чилва, было умеренно высоким, и инновационные стратегии распределения вакцины, примененные с учетом особенностей населения озера, обеспечили достаточное покрытие даже в труднодоступных сообществах. Привлечение общественности и упрощение порядка распределения были критически важны для успеха кампании. Прием пероральной холерной вакцины с нарушением в обход инструкции по применению (без соблюдения холодовой цепи) оказался эффективной стратегией для обеспечения высокого покрытия в труднодоступных сообществах. Тем не менее следует продолжить мониторинг покрытия и эффективности как в краткосрочной, так и в долгосрочной перспективе.

## Introduction

**Fig. 1:**
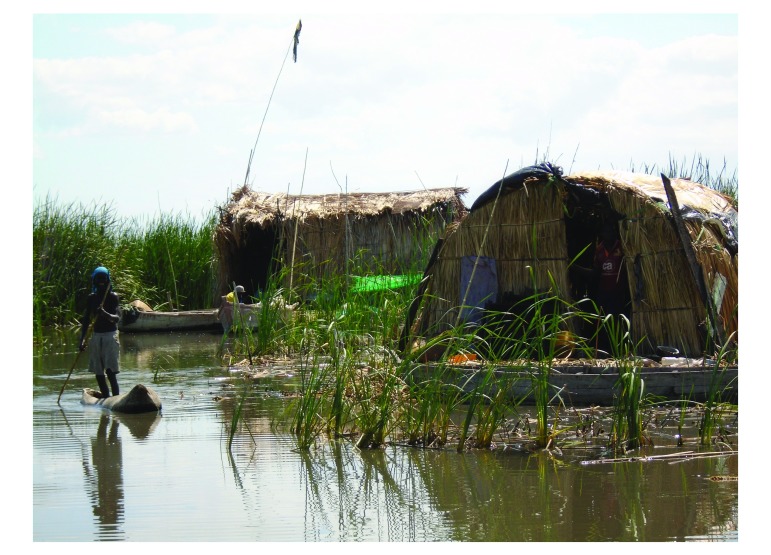
*Zimbowera*, Lake Chilwa, Malawi, 2016

Note: *Zimboweras* are huts built by fishermen on platforms constructed with grasses that emerge from the surface of the shallow lake. They serve as homes during the fishing season and are located a few hours from shore by paddle canoe.

In Malawi, cholera outbreaks occur frequently during the rainy season between November and March, with districts surrounding Lake Chilwa among the most affected.^1^ Particularly at risk are people living on the six islands in the lake and fishermen who settle temporarily during the fishing season in floating homes, known locally as *zimboweras*. *Zimboweras* are huts built by fishermen on platforms constructed with grasses that emerge from the surface of the shallow lake (Fig. 1). They are typically a few hours from shore by paddle canoe. The inhabitants of *zimboweras* live in unsanitary conditions and have limited access to safe drinking water or health care.^2^ As they do not store food, fishermen rely on communal facilities on larger and slightly better-equipped *zimboweras*, known as tea rooms, where they purchase foodstuffs. Tea rooms are also used for recreation and to sell catches to fish retailers.

Between December 2015 and August 2016, 1256 cholera cases were notified in the area surrounding Lake Chilwa, mainly in fishing communities, island communities and on the lake shore. Health centres in Machinga district reported the initial cases among fishermen, which includes the northern part of Lake Chilwa. The epidemic then spread to nearby Zomba and Phalombe districts.

**Fig. 2:**
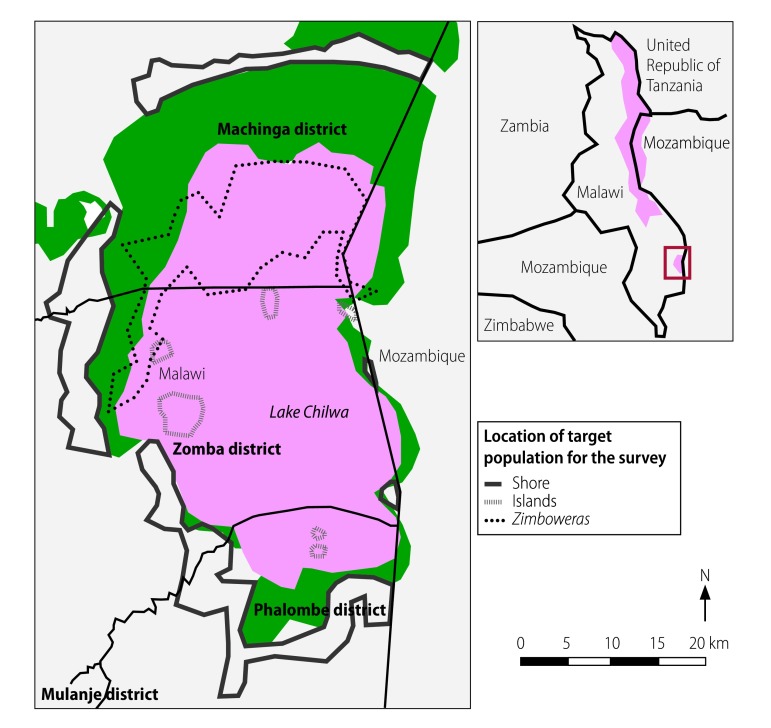
Oral cholera vaccination survey areas, Lake Chilwa, Malawi, 2016

Note: We divided the target population for the survey into three strata according to the strategy used to administer the second vaccine dose: (i) the standard strategy was used for people living on the shores of Lake Chilwa; (ii) a simplified cold-chain strategy was used for people living on islands in the lake; and (iii) an out-of-cold-chain strategy was used for fishermen living on *zimboweras*, which are temporary floating homes built for the fishing season.

In response, the Malawian Ministry of Health, supported by the World Health Organization (WHO) and international partners, including Agence de Médecine Préventive and Médecins sans Frontières, launched a two-dose cholera vaccination campaign in addition to strengthening surveillance, case management and water and sanitation improvements. The campaign targeted 80 000 people, who comprised all residents of villages located less than approximately 2 km from the lake shore, all residents on the islands and the *zimboweras* fishermen communities (Fig. 2). Patients from neighbouring Mozambique were also treated in a health centre close to the border, but there was no formal collaboration with Mozambican health authorities on vaccinating people on the eastern lake shore.

The first round of the vaccination campaign took place between 16 and 20 February 2016 and the second round, between 8 and 11 March 2016. An oral cholera vaccine was used: Shanchol^TM^ (Shantha Biotechnics, Hyderabad, India). All individuals received their first dose at vaccine distribution sites via the standard method (i.e. directly observed vaccination). The second dose was also administered in this way in shore communities, whereas two innovative strategies were used on the islands and *zimboweras*. On the islands, the strategy involved two simplifications. First, vaccine vials were entrusted to community leaders in a simplified cold chain, which alleviated the logistical needs of preparing a second round. Second, household heads were given the opportunity to collect vials for all household members to administer at home. However, the second dose could alternatively be given by directly observed vaccination if family members attended a vaccine distribution site. The *zimbowera* fishermen also received the first dose by directly observed vaccination, but were given the second dose in zipper storage bags. Fishermen were instructed to keep the bags in their *zimboweras* and to take the second dose by themselves 14 days later. Nineteen of the most frequented tea rooms were used as distribution sites. The vaccination campaign was advertised through community health workers, zone and district executive committees, schools and radio stations. Megaphones were used to remind fishermen to take the second dose.

**Fig. 3:**
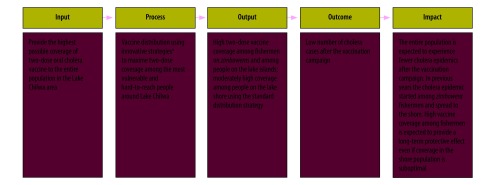
Oral cholera vaccination programme evaluation, Lake Chilwa, Malawi, 2016

^a^ The innovative strategies involved distributing the second vaccine dose using a simplified cold chain on the islands and using out-of-cold-chain self-administration on *zimboweras*, which are temporary floating homes built for the fishing season.

Implementing timely oral cholera vaccine campaigns in response to outbreaks remains challenging.^3^^,^^4^ Several reactive campaigns, with good coverage and acceptability, have been documented in recent years.^3^^,^^5^^–^^7^ However, these campaigns were conducted in relatively stable populations that could be reached using traditional mass vaccination strategies. Our campaign around Lake Chilwa was the first to use strategies involving self-administration or simplified delivery of the second dose. We expected these innovative strategies to maximize coverage with two vaccine doses among the most vulnerable and hard-to-reach populations in the area. High vaccination coverage among fishermen should reduce the risk of future epidemics, not only in the *zimbowera* community, but also in the entire population around Lake Chilwa (Fig. 3).

The aims of this study were to estimate vaccination coverage following the cholera vaccine campaign in the Lake Chilwa area in February and March 2016, to identify reasons for non-vaccination and to assess satisfaction with the innovative vaccine delivery strategies used. We focused on evaluating strategies that could be used in future in similar hard-to-reach populations.

## Methods

The study population comprised individuals older than 1 year, including pregnant women, the same as the target population of the oral cholera vaccine campaign. We divided the population into three strata according to the vaccination strategy adopted: (i) approximately 72 000 people living in villages located within 2 km of the shore of Lake Chilwa who were vaccinated using the standard strategy; (ii) approximately 6700 people living in villages located on islands in the lake who were vaccinated using a simplified cold-chain strategy; and (iii) approximately 6000 fishermen living on *zimboweras* who were vaccinated using an out-of-cold-chain strategy (Fig. 2). Study participants were selected using a two-stage, cluster sampling process, with sampling procedures adapted to the information available for each stratum. In the shore stratum, the first household in each cluster was selected using spatial random sampling based on Google Earth satellite images, as previously described.^6^ Thereafter, the nearest four houses were surveyed to give a total of five households per cluster. In the island stratum, the first household in each cluster was randomly selected using a list of households from a census conducted before the vaccination campaign. Again, the four nearest houses were also surveyed. In *zimbowera* communities, we exhaustively mapped tea rooms before the survey and established the average number of fishermen who visited each: the average ranged from 5 to 100 fishermen per day. Clusters of five fishermen were selected in proportion to the number of daily visits at each tea room. Of 60 tea rooms, 46 were selected: the number of fishermen interviewed at each ranged from 5 to 15.

All eligible individuals living in each selected household were interviewed. A household was defined as a person or a group of related or unrelated people who had lived together in the same dwelling unit for at least two weeks. Young children were interviewed together with their caregivers to ensure accurate responses. If a household member was not at home at the time of the survey, the interviewer returned later that day to interview the absentee. For people living in *zimboweras*, interviewers arrived at the tea rooms as early as possible in the morning and interviewed fishermen in order of their arrival until the required number was reached.

**Fig. 4:**
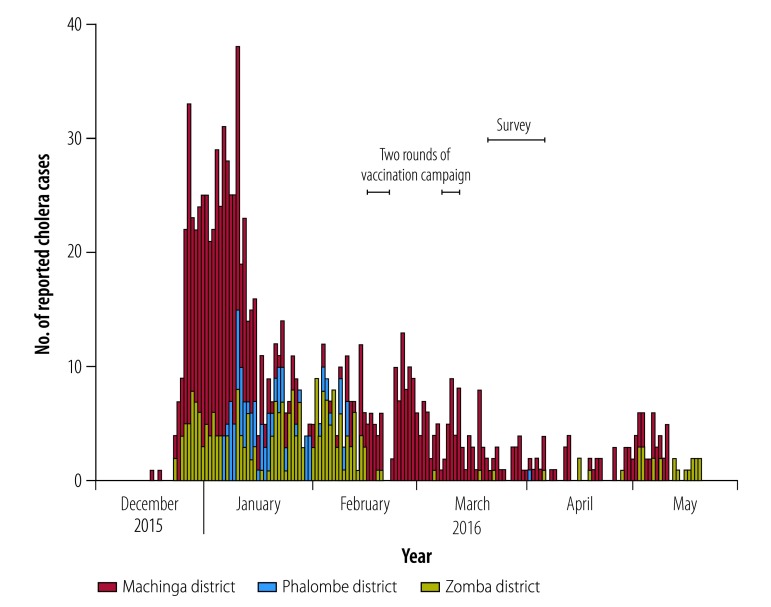
Reported cholera cases, by district and time, Lake Chilwa, Malawi, 2016

Note: We carried out the oral cholera vaccination programme in two rounds, from 16 to 20 February 2016 and from 8 to 11 March 2016, respectively, and the vaccination coverage survey took place between 21 March and 6 April 2016.

The survey was carried out between 21 March and 6 April 2016, shortly after the second vaccination round (Fig. 4). Using paper questionnaires, we collected data on: (i) demographic characteristics, such as age, sex and household size; (ii) the number of oral cholera vaccine doses taken; (iii) the date of vaccination; (iv) the main reasons for non-vaccination; (v) the presence and type of any reported adverse events following immunization; and (vi) knowledge of oral cholera vaccination. The number of vaccine doses received was determined from vaccination cards or the individual’s recall. We also collected information on the acceptability of the novel vaccination strategies on the islands and *zimboweras*. Three teams, comprising four surveyors and one supervisor, did the survey. All underwent two days’ training. Surveyors used a field manual and local calendars, to make it easier for participants to recall dates, during the standardized data collection.

### Statistical analysis

For the shore and island strata, we calculated sample sizes to obtain sufficiently precise estimates in the age groups 1 to 4, 5 to 14, and 15 or older years. In practice, sample sizes were based on the 1 to 4-year-old age group, which was the smallest age group in the population. Assuming the proportion expected to receive two doses was 70%, an α error of 5%, a precision of 10% and design effect of 3, the necessary sample size was 242 children in this age group. The further assumption of incomplete data or refusal rate of 10% increased the required sample size to 270 children. According to the 2010 Malawi Demographic and Health Survey,^8^ there were 0.8 children aged 1 to 4 years per household. Consequently, we estimated that 340 households (i.e. 68 clusters of five households) needed to be interviewed on shore. For the island population, finite population sampling correction resulted in a lower sample size of 295 households (i.e. 59 clusters of five households). For the *zimbowera* population, the only differences were: (i) the assumed incomplete data or refusal rate was 20%; and (ii) the population consisted mainly of young adults. The resulting required sample size was 295 fishermen.

We analysed the data using Stata v. 13 (StataCorp LP., College Station, United States of America), which can estimate vaccination rates and standard errors in complex survey designs. We defined vaccination coverage as the proportion of people interviewed who had been vaccinated. Given the high mobility of the target population, particularly inhabitants of *zimboweras*, we first calculated coverage estimates only for interviewed people who reported being present during the vaccination campaign and were therefore eligible for vaccination. In addition, we calculated second coverage estimates by including interviewed people who arrived in the location after the vaccination campaign. We calculated estimates for each vaccine dose taken. A similar approach was used to calculate the frequency of adverse events following immunization. We report other variables, especially those relating to knowledge of cholera vaccination, using descriptive statistics. The survey was approved by the National Health Sciences Research Committee of Malawi and by the *Comité de Protection des Personnes* in Saint-Germain-en-Laye, France. Verbal consent was obtained from all participants.

## Results

In total, the teams interviewed 1477 people on the lake shores, 1153 on the islands and 295 on *zimboweras*. In the *zimboweras*, 284 of the 295 (96.3%) were men, 291 (98.6%) were aged 15 years or older and 59 (20.0%) arrived after the second vaccination round (Table 1). The median age of the participants on the lake shores was 14 years (interquartile range, IQR: 7–29), on the islands 18 years (IQR: 8–30) and on the *zimboweras *was 30 years (IQR: 23–38).

**Table 1:** Participants’ characteristics, survey of oral cholera vaccine coverage, Lake Chilwa, Malawi, 2016

Demographic characteristic	No. of participants (%)^a^ by area of residency^b^		
	Shore (*n* = 1477)	Islands (*n* = 1153)	*Zimboweras* (*n* = 295)
**Arrival date at interview location**			
Before 1 January 2016	1443 (97.7)	947 (82.1)	141 (47.8)
Between 1 January 2016 and first vaccine dose distribution	4 (0.3)	40 (3.5)	38 (12.9)
Between first and second vaccine dose distribution	4 (0.3)	119 (10.3)	57 (19.3)
After second vaccine dose distribution	8 (0.5)	9 (0.8)	59 (20.0)
Did not know or remember	18 (1.2)	38 (3.3)	0 (0.0)
**Sex^c^**			
Female	779 (53.1)^d^	554 (48.2)^d^	11 (3.7)
Male	689 (46.9)^d^	596 (51.8)^d^	284 (96.3)
**Age, years^e^**			
1–4	222 (15.1)^f^	159 (13.8)	1 (0.3)
5–14	516 (35.0)^f^	346 (30.0)	3 (1.0)
≥ 15	735 (49.9)^f^	648 (56.2)	291 (98.6)

^a^ All values represent absolute numbers and percentages unless otherwise stated.

^b^ Survey participants lived either on the shores of Lake Chilwa, on islands in the lake, or on *zimboweras*, which are temporary floating homes built for the fishing season.

^c^ Data on the sex of 9 people on the shore and 3 on the islands were missing.

^d^ The figures represent percentages of the number of people for whom sex data were available.

^e^ Data on the age of 4 people on the shore were missing.

^f^ The figures represent percentages of the number of people for whom age data were available.

Overall, 1153 of the 1451 (79.5%) people on the shore who were present during the vaccination campaign received at least one dose, as did 1098 of the 1106 (99.3%) present on the islands and 200 of the 236 (84.7%) present on *zimboweras*. Additionally, coverage with two doses was 53.0% (769/1451) on shore, 91.3% (1010/1106) on the islands and 78.8% (186/236) on *zimboweras* (Table 2). Coverage with at least one dose in those aged 15 years or older on the islands was similar (99.0%, 613/619) to that in those younger than 15 years but, on shore, it was significantly lower, at 74.0% (534/722) versus 85.0% (617/726) in the younger age group (*P* < 0.001). We found no difference in coverage between the sexes in any of the three strata (Table 2). Calculating vaccination coverage for people present during the survey did not result in any significant change in estimated coverage either on the shore or islands, whereas, on *zimboweras*, coverage was lower: 72.5% (214/295) for at least one dose and 67.5% (199/295) for two doses (Table 2). The percentage of people who took the first dose during the first round, but did not take the second dose (i.e. the drop-out rate) was 25.9% (268/1035) on shore, 6.7% (73/1083) on the islands and 7.0% (14/200) on *zimboweras*. The drop-out rate was particularly high (33.3%; 159/477) on the shore in Machinga district. The most frequently reported reason for not taking the vaccine was absence during the campaign in all three strata. Another common reason was that the vaccine was not available at the vaccination post (Table 3).

**Table 2:** Oral cholera vaccine coverage, by area of residency, Lake Chilwa, Malawi, 2016

Cholera vaccine doses received**^a^**	Area of residency^b^													
	Shore					Islands					*Zimboweras*			
	No. surveyed	People vaccinated		*D_eff_*		No. surveyed	People vaccinated		*D_eff_*		No. surveyed	People vaccinated		*D_eff_*
		No.	% (95% CI)				No.	% (95% CI)				No.	% (95% CI)	
**People present during the vaccination campaign**														
At least one dose (recall or card)	1451	1153	79.5 (72.3–85.1)	9.1		1106	1098	99.3 (98.2–99.7)	1.7		236	200	84.7 (78.0–89.7)	1.5
At least one dose (card only)	1451	1062	73.2 (65.7–79.5)	8.9		1106	1053	95.2 (92.2–97.1)	3.4		236	132	55.9 (43.6–67.6)	3.5
Two doses (recall or card)	1451	769	53.0 (45.2–60.7)	9.0		1106	1010	91.3 (87.4–94.1)	3.7		236	186	78.8 (69.8–85.7)	2.2
At least one dose (recall or card), by age in years^c^														
1–14	726	617	85.0 (76.9–90.6)	6.6		487	485	99.6 (97.0–99.9)	2.0		3	2	66.7 (14.2–96.0)	NA
≥ 15	722	534	74.0 (67.1–79.8)	6.6		619	613	99.0 (97.9–99.6)	1.0		233	198	85.0 (78.5–89.8)	NA
At least one dose (recall or card), by sex^d^														
Female	763	618	81.0 (73.8–86.6)	5.1		532	530	99.6 (97.3–99.9)	2.0		9	9	100.0 (NA)	NA
Male	679	528	77.8 (69.9–84.0)	4.9		571	565	98.9 (97.7–99.5)	0.9		227	191	84.1 (77.3–89.2)	1.5
**People present during the survey**														
At least one dose (recall or card)	1477	1167	79.0 (71.9–84.7)	9.1		1153	1136	98.5 (97.1–99.2)	1.9		295	214	72.5 (63.9–79.8)	2.3
At least one dose (card only)	1477	1073	72.6 (65.1–79.1)	9.1		1153	1091	94.6 (91.3–96.7)	3.9		295	136	46.1 (34.7–57.9)	4.1
Two doses (recall or card)	1477	779	52.7 (45.0–60.4)	9.0		1153	1046	90.7 (86.6–93.7)	4.2		295	199	67.5 (58.2–75.5)	2.5

CI: confidence interval; *D_eff_*: design effect; NA: not applicable.

^a^ People either recalled the number of vaccination doses received or presented their vaccination cards.

^b^ Survey participants lived either on the shores of Lake Chilwa, on islands in the lake or on *zimboweras*, which are temporary floating homes built for the fishing season.

^c^ Data on the age of 4 people on the Lake shore (2 vaccinated and 2 not vaccinated) were missing.

^d^ Data on the sex of 9 people on shore and 3 on the islands were missing.

**Table 3:** Reasons for not receiving oral cholera vaccine, by area of residency, Lake Chilwa, Malawi, 2016

Reason for non-vaccination	No. of survey respondents (%) by area of residency^a^		
	Shore	Islands	*Zimboweras*
Absent, ill or at work	111 (33.7)	9 (47.3)	47 (55.3)
Vaccine not available when visiting vaccination site	71 (21.6)	0 (0.0)	13 (15.3)
Unaware of vaccination campaign	33 (10.0)	1 (5.3)	12 (14.1)
Unaware of need for cholera vaccination	29 (8.8)	0 (0.0)	2 (2.4)
Vaccination post too far away	11 (3.3)	0 (0.0)	0 (0.0)
Vaccinators absent when visiting vaccination site	9 (2.7)	0 (0.0)	2 (2.4)
Aware of campaign but not of location or time of vaccination	7 (2.1)	0 (0.0)	4 (2.2)
Vaccination not authorized by head of family	9 (2.7)	0 (0.0)	0 (0.0)
Lack of confidence in vaccination	8 (2.4)	0 (0.0)	0 (0.0)
Fear of side-effects or influenced by rumours that cholera vaccine is harmful	5 (1.5)	1 (5.3)	0 (0.0)
Unaware of being eligible for vaccination	3 (0.9)	1 (5.3)	0 (0.0)
Long waiting time at vaccination site	3 (0.9)	0 (0.0)	0 (0.0)
Religious reasons	3 (0.9)	0 (0.0)	0 (0.0)
Caretaker not available to bring child or other family member	1 (0.3)	0 (0.0)	0 (0.0)
Other	26 (7.9)	7 (36.8)	5 (5.9)
**Total**	**329 (100)**	**19 (100)**	**85 (100)**

^a^ Survey participants lived either on the shores of Lake Chilwa, on islands in the lake or on *zimboweras*, which are temporary floating homes built for the fishing season.

On the islands, 54 of the 1046 individuals (5.2%) who received a second dose reported receiving it from a family member who had collected the vial from a vaccination site. Of these 54, 51 (94.4%) found this mode of delivery practical and convenient (Table 4). Nevertheless, most people on the islands (i.e. 938 individuals, 89.7%) went to a vaccination post for their second dose (details of the remaining locations are available from the corresponding author). Of the 176 fishermen on *zimboweras* who reported self-administering the second dose, 6 (3.4%) took it less than 13 days after the first dose, 13 (7.4%) took it 13 days after exactly, 117 (66.5%) took it between 14 and 21 days after and 20 (11.4%) took it 22 days or more after. The longest delay was 46 days. For 20 of the 176 fishermen (11.4%), it was not possible to determine the time between the two doses precisely. Of the 176, 124 (70.5%) found self-administration to be practical and convenient, whereas 17 (9.7%) reported that self-administration was complicated or that they did not like it (Table 4). The reasons for not liking self-administration were: (i) fear of losing the vial (8 fishermen); (ii) not wanting to be responsible for taking the vaccine (5 fishermen); and (iii) fear of forgetting to take it (4 fishermen).

**Table 4:** Vaccinees opinions of novel strategies for administering the second oral cholera vaccine dose, Lake Chilwa, Malawi, 2016

Vaccinees opinion of strategy	No. of survey respondents^a^ (%) by administration strategy for second vaccine dose	
	Self-administration after a family member collected the vial from a vaccination site (islands)	Self-administration 2 weeks after receiving the vial during distribution of the first dose (*zimboweras*)
It was practical and convenient	51 (94.4)	124 (70.5)
It was complicated	0 (0.0)	11 (6.2)
Did not like it	2 (3.7)	6 (3.4)
No response	1 (1.9)	35 (19.9)
**Total**	**54 (100)**	**176 (100)**

^a^ Survey respondents lived in hard-to-reach areas, either on islands in Lake Chilwa or on *zimboweras*, which are temporary floating homes built for the fishing season.

## Discussion

Our survey found that the novel oral cholera vaccine distribution strategies were associated with a high level of coverage and were widely accepted by survey participants. These strategies simplified the logistics of delivering the vaccine and were more readily accepted by vaccinees than traditional directly observed vaccination: high coverage was achieved in communities considered difficult to reach, such as fishermen living on *zimboweras* and people on the islands. Drop-out rates were lower in these areas than on shore and were lower than achieved in other oral cholera vaccine campaigns that used traditional delivery strategies (e.g. 15.3% in Guinea in 2012 and 9.6% in Haiti in 2013).^6^^,^^9^

Concerns reported by fishermen about self-administration of the second dose related mainly to fear of losing the vial or forgetting to take the dose. The latter concern was addressed by a publicity campaign that was carried out when the second dose was due to be taken and which again used the existing network of tea-room managers. Fear of losing the vial was justified because fishermen preferred to keep vials in their pockets rather than in *zimboweras,* which are frequently shared with unrelated individuals. Nevertheless, the drop-out rate among fishermen was low, which indicated good compliance. This is remarkable considering that most fishermen were young men, who are generally the most difficult to target in vaccination campaigns.^6^^,^^10^

The survey showed that coverage among *zimbowera* fishermen varied markedly between those who were present during the vaccination campaign and those who arrived during the survey, two weeks after the campaign. This variation is a clear indication of the high mobility of this population. Although some fishermen were vaccinated on shore or on an island before moving to a *zimbowera*, others may not have had the opportunity, especially if they came from villages not covered by the campaign. This is the most probable reason for the small rebound in cholera cases recorded in May 2016 at health centres in Machinga and Zomba districts (Fig. 4). Another oral cholera vaccine campaign was carried out in November 2016 in *zimboweras* and villages within 25 km of the lake shore, it partially overlapped the area covered by the campaign in February and March 2016. The second campaign provided an opportunity for vaccination to fishermen who were not vaccinated in the earlier campaign.^11^ A complementary way of maintaining adequate coverage in this highly mobile population could be to distribute vaccine routinely at lake entry points.

On the islands, the strategy used to distribute the second dose simplified logistics and home-based administration was liked by those who used it. Nevertheless, most people on the islands preferred to be vaccinated at vaccination points. An anthropological survey carried out in parallel suggested that the innovative strategy was not well understood by some community leaders and, thus, communication with the community was poor.^12^

The moderate level of coverage achieved on the lake shore might be explained by two factors. First, it is likely that residents of neighbouring villages outside target areas also came to vaccination sites, thereby reducing the stocks available for the target population. Second, we cannot exclude the possibility that the target population on the shore had been underestimated, which may have resulted in vaccine shortages at some sites. These two factors should be considered in future campaigns in open settings.

The evaluation methods used in this study were relatively complex. Different sampling procedures were used in each stratum and fishermen communities were sampled by carrying out a census of tea room attendance. We are confident that the sample of fishermen in our survey was representative of the *zimbowera* population, because we mapped 60 tea rooms before the survey, much more than the 19 used for vaccination, and because fishermen were known to attend tea rooms regularly. Nevertheless, possible selection biases cannot be excluded. For example, fishermen’s attendance at a tea room may have been affected by the distance of their *zimboweras* from the tea room or by their fishing activities. Moreover, although we tried to list all tea rooms around the lake, it is possible that we missed some small tea rooms. Another limitation was that we ascertained vaccination status from both oral reports and vaccination cards. Nevertheless, most people in the three strata had cards, though the percentage was lower among fishermen.

Finally, design effects were higher than anticipated, particularly on the shore. This reflected the high heterogeneity in vaccination coverage between clusters, which was under 30% in some clusters and over 90% in others. An in-depth analysis of the data found that no survey respondent reported being vaccinated in three clusters in Zomba district that were geographically close to each other. When these three clusters were removed from the analysis, the design effect dropped from 9.1 to 6.7. Nevertheless, estimated vaccination coverage among adults on shore, both overall and in different age and sex groups, tended to be lower than in the other two strata, a problem that has already been documented in previous vaccination campaigns.^6^

The off-label use in this campaign was based on the vaccine’s documented thermal stability.^13^^,^^14^ Given limited resources, the health ministry decided it was important to implement self-administration of vaccine outside of a cold chain in a hard-to-reach and highly mobile population. In addition to increasing coverage, self-administration of the second dose improved the campaign’s cost–effectiveness by markedly reduced operational costs, such as the cost of renting boats.^15^ Considering the advantages of these novel strategies, it would be helpful if oral cholera vaccine producers could provide thermal stability data in accordance with WHO’s guidelines^16^ and could apply for controlled temperature chain licences. This would enable the regulated use of these strategies, as has been successfully implemented for meningococcal A conjugate vaccine.^17^^,^^18^

In conclusion, the oral cholera vaccination campaign in Lake Chilwa, which was implemented in three different social and geographical contexts, achieved fairly high coverage despite major logistical challenges. The two novel strategies involved should be considered for use in hard-to-reach populations in both reactive and preventive oral cholera vaccine campaigns.
